# Towards Design Optimization of Compliant Mechanisms: A Hybrid Pseudo-Rigid-Body Model–Finite Element Method Approach and an Accurate Empirical Compliance Equation for Circular Flexure Hinges

**DOI:** 10.3390/biomimetics9080471

**Published:** 2024-08-03

**Authors:** Masoud Kabganian, Seyed M. Hashemi

**Affiliations:** Department of Aerospace Engineering, Toronto Metropolitan University (Formerly Ryerson University), Toronto, ON M5B 2K3, Canada; mkabganian@torontomu.ca

**Keywords:** compliant mechanisms, circular flexure hinge, Pseudo-Rigid-Body Model (PRBM), finite element method (FEM), safety factor (SF), empirical polynomial equation, flexure hinges, torsional stiffness, rotational compliance, flexibility, mechanical design, optimization, geometric cantilever bar, four-bar mechanism, multi-bar configuration, *ABS plastic*, aluminum alloy 7075

## Abstract

Innovative designs such as morphing wings and terrain adaptive landing systems are examples of biomimicry and innovations inspired by nature, which are actively being investigated by aerospace designers. Morphing wing designs based on Variable Geometry Truss Manipulators (VGTMs) and articulated helicopter robotic landing gear (RLG) have drawn a great deal of attention from industry. Compliant mechanisms have become increasingly popular due to their advantages over conventional rigid-body systems, and the research team led by the second author at Toronto Metropolitan University (TMU) has set their long-term goal to be exploiting these systems in the above aerospace applications. To gain a deeper insight into the design and optimization of compliant mechanisms and their potential application as alternatives to VGTM and RLG systems, this study conducted a thorough analysis of the design of flexible hinges, and single-, four-, and multi-bar configurations as a part of more complex, flexible mechanisms. The investigation highlighted the flexibility and compliance of mechanisms incorporating circular flexure hinges (CFHs), showcasing their capacity to withstand forces and moments. Despite a discrepancy between the results obtained from previously published Pseudo-Rigid-Body Model (PRBM) equations and FEM-based analyses, the mechanisms exhibited predictable linear behavior and acceptable fatigue testing results, affirming their suitability for diverse applications. While including additional linkages perpendicular to the applied force direction in a compliant mechanism with *N* vertical linkages led to improved factors of safety, the associated increase in system weight necessitates careful consideration. It is shown herein that, in this case, adding one vertical bar increased the safety factor by $\frac{100}{N}$ percent. The present study also addressed solutions for the precise modeling of CFHs through the derivation of an empirical polynomial torsional stiffness/compliance equation related to geometric dimensions and material properties. The effectiveness of the presented empirical polynomial compliance equation was validated against FEA results, revealing a generally accurate prediction with an average error of 1.74%. It is expected that the present investigation will open new avenues to higher precision in the design of CFHs, ensuring reliability and efficiency in various practical applications, and enhancing the optimization design of compliant mechanisms comprised of such hinges. A specific focus was put on *ABS plastic* and aluminum alloy 7075, as they are the materials of choice for non-load-bearing and load-bearing structural components, respectively.

## 1. Introduction

Morphing wing designs based on Variable Geometry Truss Manipulators (VGTMs) [1] and articulated helicopter robotic landing gear (RLG) systems [2] are examples of innovative aerospace designs inspired by nature, which have drawn a great deal of attention from industry. On the other hand, compliant mechanisms, joint-free systems using flexure/torsion hinges and hybrid topology to create motion, have become increasingly popular due to their advantages over conventional mechanisms *consisting of rigid links connected through revolute joints*. These advantages include, but are not limited to, enhanced accuracy and reliability, along with lower wear, backlash, maintenance, and weight, accomplished through topology optimization and advanced manufacturing methods. These characteristics are valuable for applications where the system is difficult to access, weight reduction is of prime importance, and variable-geometry structures are involved, such as morphing wings and landing gear systems. Zhu et al. (2023) [3] showed that these flexible and compliant mechanisms provide significant advantages in aerospace engineering, potentially leading to lighter systems, improved lift, reduced fuel consumption, and minimized noise, especially in morphing wings [3]. Furthermore, the exploitation of flexure hinges offers potential for enhancing landing safety in articulated helicopter robotic landing gear (RLG) systems through mitigating the risk of joint failures, as highlighted in the study by Manivannan et al. (2013) [2]. In a more recent study, Kim et al. (2023) [4] demonstrated that the use of locking mechanisms effectively eliminates landing failures that may occur with non-locking landing gear configurations, emphasizing the importance of flexure hinges versus traditional joints [4].

Compliant mechanisms represent a groundbreaking departure from the conventional rigid-body mechanisms by offering a joint-free solution. Instead of relying on traditional joints and bearings, compliant mechanisms harness the flexibility of flexure hinges to facilitate motion. The flexure hinge is a special type of mechanical component that allows restricted angular movement by utilizing the material’s micro-elastic properties. This innovative approach opens the door to more efficient designs capable of delivering higher precision and accuracy, particularly in fields like robotics and machine tools. Moreover, the inherent flexibility of flexure hinges enhances reliability and durability, especially when compared to their rigid counterparts. As also mentioned above, in comparison with their conventional counterparts, compliant mechanisms also eliminate or reduce issues such as friction, noise, lubrication, wear, and backlash [5], and might be selected for a specific application due to their various benefits. As a result of these advantages, compliant mechanisms have been gaining popularity, rendering them well-suited for a wide array of applications.

The primary benefits of compliant mechanisms fall into two categories: economic savings (fewer parts, reduced assembly time, and simplified manufacturing processes), and enhanced performance (greater reliability and accuracy, and reduced wear, weight, and maintenance) [6]. They enable precise motion by minimizing or eliminating wear and backlash. In addition, compliant mechanisms feature fewer or no joints, such as pins and rotary bearings, reducing friction and the necessity for lubrication. These properties are particularly valuable for applications where maintenance access is challenging, weight reduction is critical, or they are operating in harsh environments, including high-temperature, hazardous areas, and space, where lubricants may outgas or evaporate [6]. Compliant mechanisms have also been successfully miniaturized for microelectromechanical system (MEMS) applications. Mechanical displacement amplifiers, used to increase the output displacement, are also another application of such mechanisms.

Compliant mechanisms offer a notable advantage in weight reduction compared to their rigid-body counterparts. While most of the weight savings are accomplished through topology optimization, the cost savings for these components will be realized by understanding the manufacturing processes behind producing these components in a cost-effective way. For instance, compliant mechanisms alter the design in a new manner that is flexible and strong, thereby inherently leading to simplicity of manufacturing and assembly. It is worth noting that while compliant mechanisms offer numerous advantages, they also come with several challenges and drawbacks, with one of the most significant challenges being the complexity inherent in their design and analysis.

One advantage of compliant mechanisms lies in their capacity to store energy within flexible elements. However, in certain applications, this characteristic can pose challenges. For instance, when a mechanism’s primary role involves energy transfer between members, some of the energy may be stored within the mechanism itself. Additionally, fatigue analysis becomes a more critical concern for compliant mechanisms compared to their rigid-body counterparts, as flexible components often experience cyclic loading. Furthermore, the range of motion achievable through flexure hinges is limited by the strength of their deflecting members. It also is worth noting that contrary to conventional pin joints, compliant links are unable to sustain continuous rotational motion. Moreover, compliant links subjected to prolonged stress or exposure to high temperatures can induce stress relaxation or creep in compliant links [6]. 

To effectively work with compliant mechanisms, a deep understanding of both the mechanism and flexible member analysis and synthesis, as well as their interactions in complex scenarios, is essential. For instance, the linear beam equations commonly used in traditional settings become inadequate when dealing with compliant mechanisms due to the significant deflections experienced by flexible members. To address this, nonlinear equations that accommodate geometric nonlinearities resulting from these substantial deflections must be exploited. 

**Figure 1:**
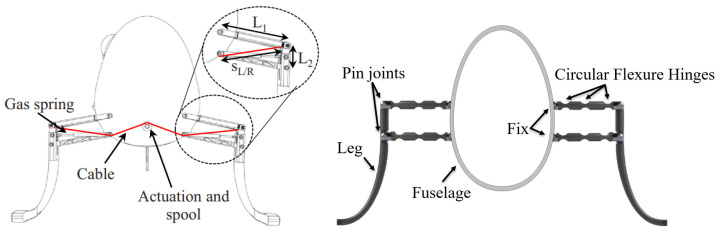
4-bar mechanism of RLG for VTOL applications: (**left**) conventional [7], (**right**) compliant.

**Figure 2:**
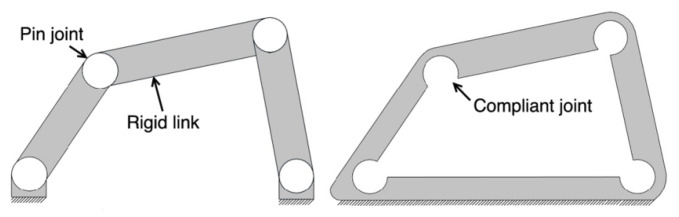
Conventional mechanism vs. compliant mechanism [8].

Historically, many compliant mechanisms were designed through trial and error, limiting their applicability to very simple systems. This approach is often not cost-effective for numerous potential applications. Despite recent efforts to streamline analysis and design processes, it is important to acknowledge that working with compliant mechanisms generally presents greater challenges than dealing with rigid-body mechanisms. A four-bar linkage is a reliable mechanism capable of efficiently transferring input motion in parallel directions, making it directly applicable to typical use cases (e.g., locking pliers and pantographs) and indirectly applicable in other mechanisms with more linkages. The conventional four-bar mechanism has also been executed in the design of a cable-driven, articulated, robotic landing gear (RLG) for vertical takeoff and landing (VTOL) [7], as shown in Figure 1 (left). The authors’ team is actively pursuing creative ways to advantageously implement flexure hinges in such aerospace applications (Figure 1, right). A four-bar mechanism and the equivalent compliant mechanism are depicted in Figure 2. 

**Figure 3:**
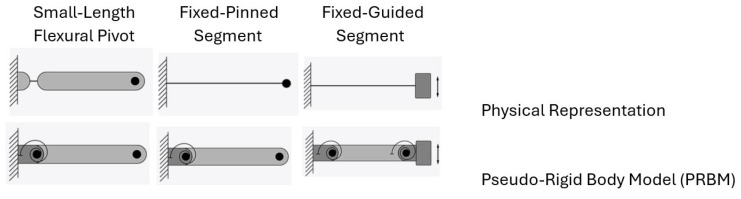
The Compliant Mechanisms (**Top**) vs. their Pseudo-Rigid-Body Model (PRBM) (**Bottom**), adapted from [11].

The finite element method (FEM) and the Pseudo-Rigid-Body Model (PRBM) play crucial roles in analyzing compliant mechanisms. FEM has been widely used in this field, providing detailed insights into the behavior of flexure members [9]. The basis for the FEM is discretization of the structure into a finite number of elements and interpolation of scalar field variables (e.g., components of deflections) at the vertices of the element (also called nodes), using what are known as shape or interpolation functions of approximation space [10]. Parametrization is an essential ingredient in the FEM-based analysis of compliant mechanisms, modeled as elastic deformable structures. The PRBM [8] simplifies the design and analysis of compliant structural elements, as shown in Figure 3 adapted from Reference [11], for three boundary conditions. A preliminary design can be created using the PRBM and then optimized to fulfil the specified design requirements. The resulting design might then be further refined using such approaches as nonlinear FEA, before being prototyped and tested.

**Figure 4:**
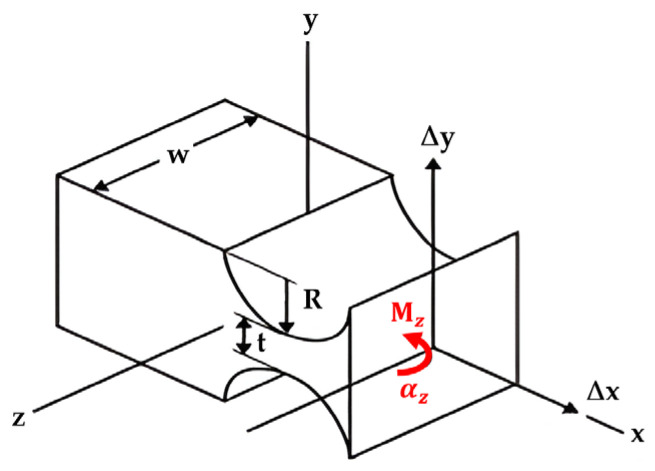
Parametric Shape of Circular Notch Hinges (adapted from [12]).

As illustrated in Figure 3 (top), the compliant mechanisms exploit some structural features (e.g., thinning, leaf hinge, notch hinge, …) to achieve the model equivalency with the PRBM (bottom). Thinning’s geometry is usually characterized by a pair of opposed cylindrical grooves. Traditionally, a leaf hinge is crafted by securing a thin plate connecting two rigid bodies, producing a thinned-out piece of section connecting them, all from the same material. Circular notch hinges (Figure 4, adapted from [12]) emerge from machining symmetrical circular patterns on each side of a solid body, forming a slender path between two rigid bodies. These notched flexures are nearly 100 percent efficient during assembly, offering an advantage over simple leaf flexure hinges. 

The integration of PRBM and FEM represents a significant advancement in compliant mechanism design, offering a more accurate framework within the conventional rigid-body mechanics paradigm. Ling et al. (2020) [13] underscored the importance of precise modeling of flexure hinges with variable cross-sections, highlighting the need for improved methodologies. Similarly, Yong et al. (2008) [12] demonstrated the presence of significant errors in analytical kinetostatic models, emphasizing the necessity for accurate empirical equations. While empirical modeling is reliable, it lacks insights for novel hinge designs and is time-consuming. Table 1, adapted from the work initiated by Yong et al. and updated by authors (as will be discussed in Section 4), summarizes the compliance/stiffness equations for specific *t*/*R* ranges proposed by different researchers, highlighting the need for more accurate modeling approaches, specifically for torsional stiffness or rotational compliance $∆\alpha_{z}/M_{z}$ of Circular-Flexure-Hinge (CFH), thereby addressing this gap by a Hybrid PRBM-FEM approach. Hence, despite challenges in accurately modeling flexure hinges, a Hybrid PRBM-FEM technique presents a promising approach to enhance the accuracy of flexure hinge modeling in compliant mechanisms.

As mentioned earlier in this paper, compliant mechanisms derive their motion from the deflection of flexure hinges, in contrast to conventional bearings and hinges. Thus, a key advantage of compliant mechanisms lies in the potential for a significant reduction in the total number of components required to accomplish a specific task. When designing a compliant mechanism, one can use the finite element method/analysis (FEM/FEA), where selecting material properties, defining geometry, and establishing boundary conditions are crucial elements of designing a compliant mechanism. The present study aims to improve the accuracy of PRBM for compliant mechanisms through the integration of the finite element method (FEM). This integration is geared towards developing an empirical polynomial torsional stiffness equation or rotational compliance, derived from FEA results, to address a range of circular flexible hinges (CFHs). The objective was to account for geometric parameter variations in the design phase, enabling the generation of highly accurate compliant mechanism designs comprised of such joints. With the primary focus set on bolstering efficiency, reliability, overall performance, and a proper safety factor (SF), a thorough analysis of the design of flexible hinges, and single-, four-, and multi-bar configurations as a part of more complex, flexible mechanisms is presented. 

The structure of the paper is as follows: Section 2, a literature review, is followed by Section 3, summarizing the PRBM (Section 3.1) and FEM (Section 3.2) methodologies used in the present study for the modeling of four-bar, cantilever-bar, and 2N-bar configurations of compliant mechanisms, and input force or moment vs. displacement, and the details of the design limits study. In Section 3.3, the outcomes of the FEM-based fatigue test are presented, offering insights into the endurance and behavior of compliant mechanisms. To address a notable gap in the literature, a novel empirical equation for rotational stiffness/compliance, in stiffness form $K_{\alpha_{z}}$, is then presented in Section 4, with an average error of 1.74%, thus contributing to the optimization of compliant mechanisms. Finally, Section 5 offers a discussion of the obtained results, followed by concluding remarks summarizing the study’s key achievements. 

## 2. Literature Review

This section presents a brief review of the literature on the topic of compliant mechanisms, with a focus on some of the key articles available in the open literature. In recent years, significant advancements have been made in the development of various types of flexure hinges, each tailored for specific applications and performance requirements. Among these, conjugated-surface flexure hinges (CSFHs) and circular flexure hinges (CFHs) are prominent due to their unique characteristics and applications. Verotti et al. (2015) [20] introduced a new CSFH, which combines a curved beam with conjugate surfaces to enhance the hinge’s performance by reducing stress in the curved beam and limiting variations in the position of the relative rotation center. Their design aimed to address some of the inherent limitations in traditional flexure hinges, such as limited motion and force transmission capabilities, and susceptibility to yielding and deformation that is dependent on the applied loads.

In comparison, traditional circular flexure hinges have been extensively studied for their applications in micro-/nano-precision mechanisms, particularly where high-resolution and high-precision motions are required. Ma et al. (2020) [21] conducted a comprehensive performance comparison of typical notched flexure hinges, including circular ones, using finite beam-based matrix modeling methods and nondimensional precision factors. This study highlighted the strengths and weaknesses of various hinge designs, providing practical guidelines for developing compliant mechanisms with excellent overall performance. Furthermore, Zhu et al. (2019) [22] utilized topological optimization to enhance the design of flexure hinges, demonstrating that starting from the topology level can yield more efficient designs with superior performance. 

Both CSFH and CFH designs play crucial roles in advancing compliant mechanism design, with the former offering improved accuracy and stress management, while the latter continues to be a reliable choice for applications demanding high positioning accuracy. Paros and Weisbord (1965) [14] were the first to introduce right circular flexure hinges (RCFHs) and developed both simplified and full design equations for calculating the compliances of flexure hinges, rooted in the material mechanics theory and idealized assumptions. The authors introduced mathematical formulations to ascertain the stiffness of single-axis and two-axis flexure hinges when subjected to combined loads, achieved through the integration of variables across the entire span of the flexure hinge. 

Smith et al. (1997) [18] presented closed-form equations specifically for calculating the mechanical compliance of monolithic flexure hinges with elliptic cross-sections. The equations, derived from modifications of those initially formulated by Paros and Weisbord, converge at certain limits to their equations for hinges with circular sections and the compliance of cantilever beams. The equation’s accuracy was confirmed through FEM simulations and experimentation. The authors claimed that the simplified equation’s error, relative to the full equation, was within 1% for hinges with *t*/*R* ratios in the range of 0.02–0.1, and within 5–12% for thicker hinges with *t*/*R* ratios in the range of 0.2–0.6. However, when compared with FEA results, both the simplified and full rotational compliance equations showed significant discrepancies, with errors up to 24% or more for *t*/*R* = 0.6. 

Ryu and Gweon (1997) [23] presented the error modeling of flexure hinges during machining using a computer-based method. Ryu et al. [24] developed a mathematical equation to optimize the design of a flexure hinge stage. Lobontiu et al. (2001) [15] conducted a systematic study on various flexure hinges, introducing closed-form solutions for corner-filleted and the conic-section flexure hinges using Castigliano’s second theorem, and validated these equations using FEA. In a similar context and in a more recent paper, Tuo et al. (2020) [25] proposed an analytical compliance model for right circle flexure hinges (RCFHs), accounting for stress concentration effects at its cross-section’s weakest point. The model, based on virtual work theory and Castigliano’s second theorem, showed relative errors within 20% for various geometric dimensions, which were further validated through FEA. Wu and Zhou (2002) [16] derived equations for general flexure hinges, producing design equations that were both precisely derived and more concise than those proposed by Paros and Weisbord. Tseytlin (2002) [17] presented a different set of equations for calculating the rotational compliance or stiffness of monolithic flexure hinges, offering an alternative to the well-known theoretical equations initially derived by Paros and Weisbord for circular hinges. The results of these equations were shown to be significantly more accurate than those of other theoretical equations. The authors claimed that circular hinges represented the worst-case error between Paros and Weisbord’s theoretical and finite element models. Wang et al. (2005) [26] conducted an extensive analysis and comparison of bow, straight beam, corner-filleted, and compound-type flexure hinges, developing theoretical models for these common flexure hinges. Yong et al. (2008) [12] conducted a comprehensive review focused on circular flexure hinge design equations, providing an overview of empirical formulations. Their study involved a meticulous comparison of compliance and stiffness equations of circular flexure hinges (CFHs) and finite element analysis (FEA) results, shedding light on the limitations of these equations at various *t*/*R* ratios. The findings of this research offer valuable guidance for choosing the accurate equations for hinge design calculations and formulates general empirical stiffness equations for both the *x* and *y* directions; however, this study did not address torsional stiffness or rotational compliance $∆\alpha_{z}/M_{z}$. The authors claimed that their presented stiffness equations exhibited errors of under 3% when compared to the FEA results across a wide range of *t*/*R* ratios, spanning from 0.05 to 0.8.

Qiu et al. (2008) [27] devised a multi-objective optimization model for flexure hinges, employing structural parameters as design variables. They focused on optimizing RCFHs without considering the influence of the shear force. Yin et al. (2011) [28] formulated a parameter optimization model specifically for shallow notch elliptic flexure hinges (EFHs). Raghavendra et al. (2010) [29] reported the design and analysis of a novel two-dimensional, compliant, monolithic, piezo-actuated microgripper utilizing flexure hinges that can perform highly precise movements, and presented a comparative study on stress and displacement, highlighting the potential for achieving high precision. Dirksen and Lammering (2011) [9] presented a study on the characterization and categorization of common planar flexure hinges with different geometries that can replace one-node hinges in optimized compliant mechanisms’ topologies. Certain analytical equations were derived in this paper, covering displacements, maximum elastic deformations, bending stiffness, center of rotation, and first natural frequencies of the flexure hinges. Deshmukh et al. (2014) [30] presented the creation of a four-bar compliant mechanism utilizing the Pseudo-Rigid-Body Model (PRBM) and demonstrated that it can achieve precise movements, which was validated by the simulation results. 

Lu et al. (2015) [31] established a stiffness model for deep-notch EFHs and conducted optimizations based on this model. Recognizing that stiffness and rotational precision in flexure hinge design are the most critical factors to be considered during flexure hinge design, this paper presents an analytical model for four types of flexure hinges, leveraging elasticity theory and the infinitesimal method to accurately calculate the above parameters. To enable a quantitative comparison of flexure hinge performance, the authors proposed and defined the hinge index. The FEA method and the experimental testing across various hinge dimensions were performed to verify the accuracy of the proposed (analytical) models. The discrepancies between the theoretical model and FEA were found to be less than 5%, and under 6% between the theoretical and experimental data. Melgarejo et al. (2018) [32] showed that the results of analytical equations to calculate the bending stiffness of thin semi-CFHs have discrepancies compared to simulation results. Henning et al. (2018) [33] presented a novel MATLAB-based computational design tool for predicting the rotational properties of notch flexure hinges used in compliant mechanisms. The authors found a good correlation between the results obtained using their analytical design tool and those from the FEM simulations. This tool is expected to be useful for the systematic and accelerated synthesis of compliant mechanisms featuring optimized flexure hinges. In a recent publication by Meyer et al. (2023) [34], the authors addressed the absence of standardized testing methods for flexure hinges by introducing novel approaches, including the tensile test, four-point bending test (FPBT), and column bending test (CBT). However, the FPBT and CBT are limited to flexure hinges with a defined transition region and constant thickness, assuming a constant curvature. Notably, these assumptions may not hold for other flexure hinge types like circular and elliptical hinges. To gain further insight into compliant mechanisms, the interested reader is referred to the recent publication by Ling et al. (2020) [13]. This review paper surveyed over 280 publications and offers guidance for those engaged in the field of compliant mechanisms on selecting suitable modeling approaches. The authors compared the conceptual kinetostatic and dynamic modeling methods for compliant mechanisms, considering both small and large deflections. The article underscores the need for innovative solutions to achieve precise modeling, with a specific call for accurate representations of flexure hinges with variable geometric dimensions and material properties, addressing the existing discrepancies among analytical models based on the geometric aspect ratio of flexure hinges.

The aim of the above literature review was to explore the potential of compliant mechanisms as a solution to industry challenges. In the present paper, the authors intend to enhance the parameterization design of compliant mechanisms by reducing errors in the design process. To this end, a hybrid approach that integrates the PRBM method with FEA is proposed to derive the torsional (or rotational) stiffness/compliance equation, which is related to geometric dimensions and material properties. The primary objective was to provide more accurate and less error-prone predictions of rotational stiffness, which would subsequently lead to optimized designs for compliant mechanisms. This, in turn, has the potential to enhance the performance of circular flexure hinge designs with a proper safety factor (SF), which, to the best of the authors’ knowledge, has not been addressed in the open literature. The design and analysis of compliant mechanisms utilizing both PRBM and FEM encompasses displacement and stress assessments, with a specific focus on comparisons between the rotational displacements and stiffnesses/compliances obtained through both methods. The critical parameters such as bending angles and maximum stress levels, serving as essential guidelines for designers, are discussed. Furthermore, a compliant mechanism featuring circular notch hinges was modeled, emphasizing cost-effective manufacturability [9]. As it will be discussed, initially, *ABS plastic* was chosen for the four-bar compliant mechanism due to its widespread use, but a transition to the stronger aluminum alloy 7075 was made after evaluating performance. As they are both as linearly elastic materials, the former is the material of choice used in the construction of non-load-bearing elements and the latter is used for load-bearing aerospace structural components. Finally, exploiting the FEM and performing a parametric study on various cantilever-bar compliant mechanisms within a range of *t*/*R* ratios, an empirical polynomial rotational compliance equation was developed, which could then be used for subsequent design phases (as it will be discussed later in this paper; *t* stands for neck thickness, and ‘*R*’ for the radius of the hinge). This effort aims to enhance the efficiency and reliability of the compliant mechanism design process, providing valuable insights for the advancement of this field. 

## 3. Methodology

The analysis of compliant mechanisms often presents significant challenges due to large deflections and complex serial–parallel configurations. Designers, especially those new to this area, may find it difficult to navigate these complexities and select an appropriate modeling approach for their specific applications. This challenge is exacerbated by the extensive knowledge in the mechanics of materials, mechanical dynamics, kinematics of mechanisms, and nonlinear mechanics required, as also emphasized in [13].

This section focuses on kinetostatic modeling of the force–deflection relationship of flexure hinges for design purposes, utilizing the Ansys-based linear finite element method and fatigue analysis. Subsequent sections will incorporate variable parameters into the modeling approach to enhance accuracy and applicability.

In the following subsections, the methodology used to comprehensively investigate and analyze compliant mechanisms is briefly explained. This hybrid methodology exploits both the Pseudo-Rigid-Body Model (PRBM) and finite element analysis (FEA) to perform modeling and simulation of various compliant mechanisms. The design and analysis of four-bar and cantilever-bar compliant mechanisms, scrutinizing their behavior under different loading conditions, is explored. The focus remained on (linearly elastic) materials like acrylonitrile butadiene styrene (ABS) plastic and aluminum alloy 7075, with a particular emphasis on safety factors, deflections behaviors in both static and fatigue analyses, and performance assessments to establish an empirical polynomial rotational compliance equation for more precise and error-resilient predictions.

### 3.1. The Pseudo-Rigid-Body Model (PRBM)

**Figure 5:**
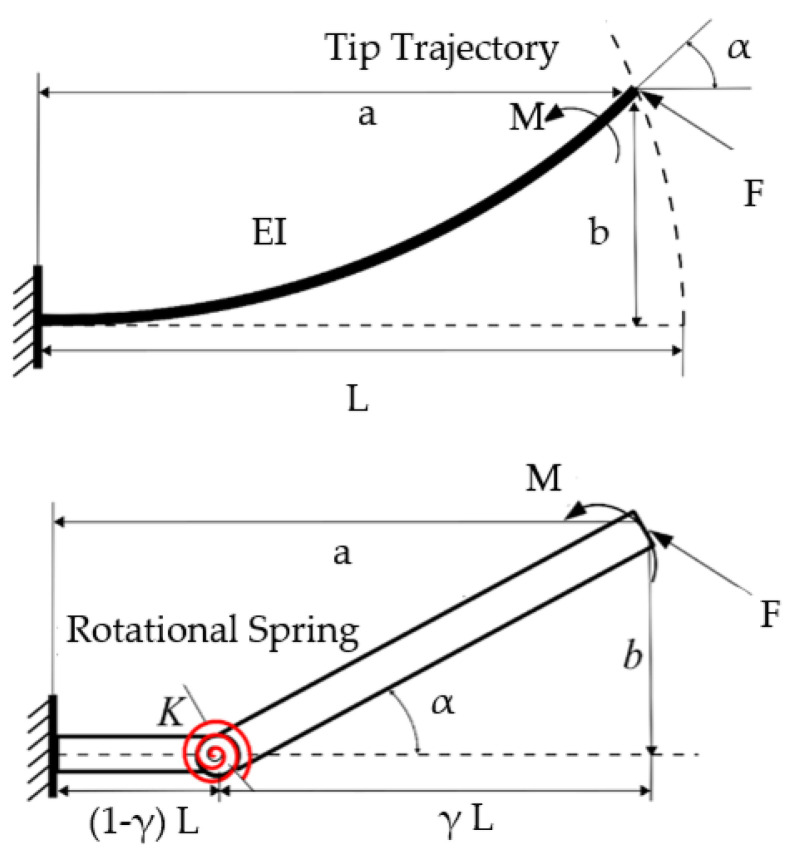
Fixed-free flexure beam and corresponding 1R PRBM (adapted from ref. [13]).

The Pseudo-Rigid-Body Model (PRBM) is a significant advancement in the field of compliant mechanism design, and focuses on various types of flexure members. PRBM research aims to bridge the gap between compliant mechanisms and conventional rigid-link mechanisms by simplifying the complex behavior of flexible elements into more predictable models [35]. Figure 5 below, adapted from ref. [13], illustrates this integration by showcasing a fixed-free flexure beam and its corresponding 1R PRBM. Despite challenges in determining characteristic pivot positions and stiffness, this model mimics the deflection paths using kinematic trajectories and approximates force–deflection relationships with a spring [6]. The beam constraint model captures load-stiffening effects and specifies the constraint behavior of flexure beams in terms of their stiffness and error motion.

The 1R model, while useful for simpler loads and configurations, may not be suitable for complex loads and intricate configurations, nor for the vibration analysis of compliant mechanisms. Instead, the PRBM typically involves performing kinematic solutions with loop closure theory or kinematic approximation, followed by static analysis based on the virtual work principle, and finally establishing a dynamic model using Lagrange’s equation [6]. For accurate modeling, assumptions play a crucial role in guiding the development of predictive models.

**Figure 6:**
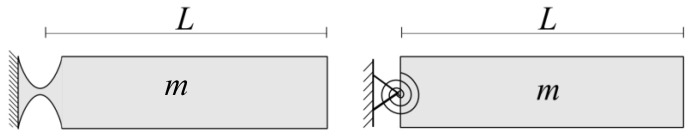
Comparison of a flexure hinge (**left**) and the equivalent discrete torsion spring model (**right**) (adapted from ref. [9]).

The assumptions include the precision of CFHs in rotation, with minimal displacement of their center of rotation compared to other flexure hinges. To validate these assumptions, insights from Dirksen and Lammering’s research (2011) [9] emphasized the significantly larger motion of the center of rotation for rectangular flexure hinges compared to CFHs, attributed to the deflection of the entire hinge length ‘*L*’. Additionally, the study underscores the relevance of natural frequency in systems with rigid bodies connected to flexure hinges under dynamic loading conditions, as illustrated in Figure 6 (left). Their conclusion aligns with the modeling of flexure hinges using discrete torsional springs, as depicted in Figure 6 (right), leveraging the rotational compliance or bending stiffness.

Yong et al. [12] provided a summary of the compliance/stiffness equations tailored for specific *t*/*R* ranges of circular flexure hinges (CFHs) proposed by different researchers, along with their minimum, maximum, and average differences. Notably, their study lacked a derivation of torsional stiffness or rotational compliance $∆\alpha_{z}/M_{z}$, primarily focusing on the translational compliances along the *x*-axis ($∆x/F_{x}$) and the *y*-axis ($∆y/F_{y}$). Figure 4 presented earlier in the Section 1 shows a visual representation of the parametric shape of the notch hinge, offering insights into its geometric characteristics. This illustration includes key parameters such as ‘*t*’ for neck thickness, ‘*w*’ for out-of-plane width (or thickness), and ‘*R*’ for the radius of the hinge. Moreover, it shows variables like $∆\alpha_{z}$, ∆x, and ∆y, representing the rotation, *x*-deformation, and *y*-deformation of the hinge, respectively, while ‘$M_{z}$’, ‘$F_{x}$’, and ‘$F_{y}$’ denote the moment and forces acting on the corresponding axes. 

Understanding the geometric attributes of the notch hinge becomes particularly relevant when considering the significance of flexure hinges in a compliant mechanism. These hinges play a crucial role in determining the elastic deformation capacity and contribute to the overall flexibility of the mechanism. Recognizing the importance of rotational movement in each hinge from a designer’s perspective and given that CFHs emphasize precise motion of the center of rotation, the authors placed a greater emphasis on their rotational compliance. Below is further discussion on the methods and equations derived by other researchers, highlighting the critical need for the accurate modeling of rotational compliance or bending stiffness to enhance the overall performance and reliability of compliant mechanisms in the parametric design optimization.

#### Rotational Compliance or Bending Stiffness

Paros and Weisbord (1965) [14] were trailblazers in developing comprehensive design equations for circular flexure hinges (CFHs). They presented two full and simplified versions of design equations to calculate the compliances of these hinges, with the so-called closed form, simplified one [14] shown in Equation (1) below:

(1) $$k_{\alpha z-Paros}=\frac{2Ewt^{2.5}}{9\pi R^{0.5}}$$

Here, *E* is the elastic modulus, and the other parameters of the notch hinge shape are illustrated in Figure 4 [14]. Equation (1) was demonstrated to generate an error within the *t*/*R* ratio range of 0.2 to 0.6, expanding to a range of 5–12% [8] for thicker hinges. Notably, when compared to the FEM results, both the simplified and full rotational compliance equations ($∆\alpha_{z}/M_{z}$) showed a substantial variance of 25% for a *t*/*R* equal to 0.6 [17]. Table 2 provides the hinge dimensions, for a *t*/*R* ratio of 0.5, as used in the analysis and designs presented in this paper. 

**Figure 7:**
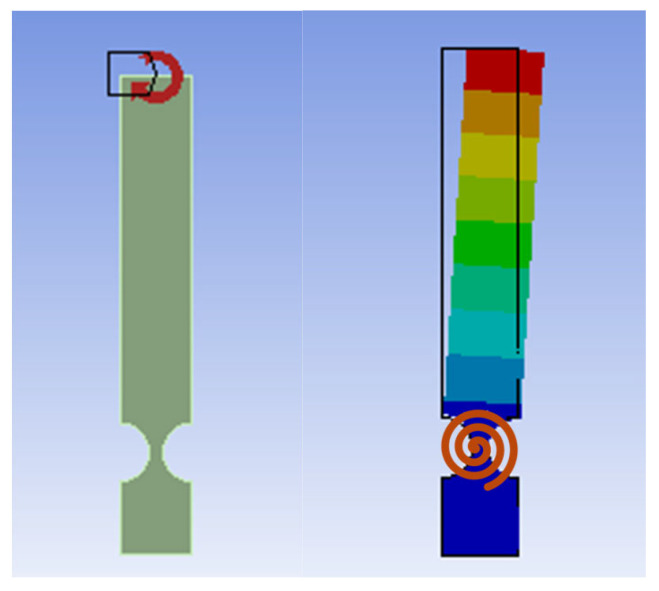
Cantilever bar with a circular flexure hinge (CFH), illustrating the bending angle resulting from the applied moment: (**left**) the undeformed system, showing the end moment, $M_{z}$; (**right**) the deformed system, with the equivalent torsional spring, $k_{\alpha z}$, superposed at the CFH.

To effectively parameterize a complex mechanism through FEA, the initial step involves breaking it down into simpler elements, such as a cantilever-bar and a four-bar mechanism. It is essential to recognize that the fixed-free bar can be regarded as a simplified version of the four-bar mechanism, providing a more straightforward analytical approach. Figure 7 below illustrates the deformation of a fixed-free, cantilever-bar, with a circular flexure-hinge, subjected to an end moment, $M_{z}$. 

**Figure 8:**
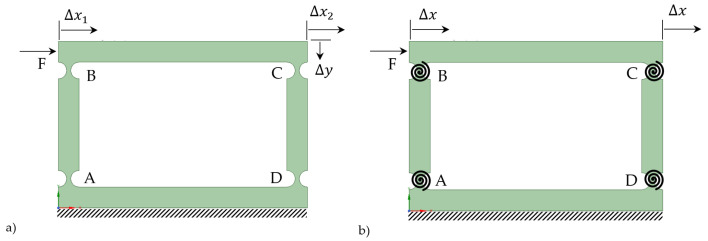
Four-bar mechanism: (**a**) compliant model; (**b**) PRBM (adapted from [27]).

As expected, applying an end moment, $M_{z}$, to a cantilever bar results in a bending angle, $∆\alpha_{z}$, as shown in Equation (2) below:

(2) $$M_{z}=k_{\alpha z}∆\alpha_{z}\;\;or\;\;k_{\alpha z}=\frac{M_{z}}{∆\alpha_{z}}\;$$

where $k_{\alpha z}$ is the equivalent torsional stiffness (see the torsional spring added in Figure 7). When conducting a Pseudo-Rigid-Body Model (PRBM) analysis of a four-bar compliant mechanism, the (flexure) hinges depicted in Figure 8a [30] are replaced with torsional springs, as demonstrated in Figure 8b. The system was confirmed to exhibit identical horizontal deflections, ${∆x}_{1}$ and ${∆x}_{2}$, i.e., 1-DOF, and near-zero undesired  ∆y [30]. This further validates the assumption of each bar acting mostly as a rigid-body mechanism with torsional springs and a horizontal displacement of ∆x, when an input force (F) is applied.

Equation (3) below shows the relationship between F and ∆x, where AB stands for the length of vertical bars AB (or DC) in a four-bar compliant mechanism (Figure 8), which was verified through an FEM.

(3) $$M_{z}=4k_{\alpha z}∆\alpha_{z}\to \;\;F(AB)=4k_{\alpha z}\frac{∆x}{AB}\;$$

In the realm of mechanical engineering and structural analysis, understanding the behavior of materials and components under various forces is paramount. One fundamental aspect of this analysis involves determining the bending angle, $∆\alpha_{z}$, of a cantilever bar in response to applied forces. This relationship can be expressed by Equation (4).

(4) $$∆x=\frac{1}{4k_{\alpha z}}F{\left(AB\right)}^{2}\;\to ∆\alpha_{z}=\arctan\left(\frac{∆x}{AB}\right)\;$$

**Figure 9:**
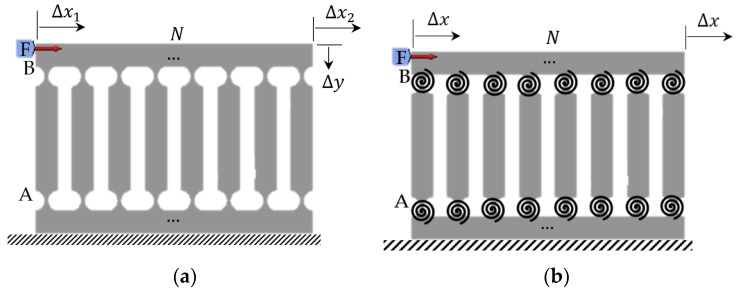
2*N*-bar mechanism: (**a**) compliant model; (**b**) PRBM.

Continuing with the four-bar PRBM analysis, a general PRBM of parallel compliant mechanisms can now be investigated. Consider a 2*N*-bar compliant mechanism consisting of *N* vertical linkages with two flexure hinges at each linkage, *N* − 1 couplers, and one base (fixed) bar. In this case, the 2*N* flexure hinges are replaced with 2*N* torsional springs, as depicted in Figure 9. Like the previous 4-bar system, applying an input force, *F*, results in a horizontal displacement of ∆*x.* The system exhibits near-zero undesired vertical displacement ∆y. 

It is noteworthy that, as per Equation (5) below, an *N*-times larger input moment/force is needed for a 2*N*-bar compliant mechanism to achieve the same horizontal displacement of ∆x as in the four-bar system (also refer to the Section 3.3.1).

(5) $$M_{z}=2Nk_{\alpha z}∆\alpha_{z}\to \;\;F(AB)=2Nk_{\alpha z}∆x/AB\;$$

In conclusion, the equations presented above allow for the derivation of force and/or moment using the PRBM method, enabling the calculation of displacement ∆x or bending angle $∆\alpha_{z}$, and vice versa. These displacement or angle values are crucial parameters for evaluating the structural response and behavior of compliant mechanisms under different loading conditions. Understanding the relationship between the applied moment, forces, displacement, and bending angle within the PRBM framework is essential for the comprehensive analysis and design of compliant mechanisms. Simplifying the analysis process through this substitution facilitates parametric calculations and optimization design, which can be accurately achieved by integrating FEA and validating the results.

### 3.2. Finite Element Analysis Using Ansys

The finite element analysis (FEA) of this compliant mechanism was conducted using the academic version of Ansys (R2 and R3) Workbench. The primary objective of this analysis was to evaluate the maximum stresses experienced by the mechanism. Additionally, the authors aimed to ensure that the stresses within the flexural members remained below the yield stress, preventing any transition to the plastic phase. To this end, *ABS plastic* (with a Young’s Modulus of E=2.39 GPa, and Poisson ratio of *ν* = 0.399) was first utilized as the primary material for this mechanism. Once the performance of the compliant mechanism was evaluated, the *ABS plastic* was replaced with aluminum alloy 7075 for the next phase of the design. This high-strength aluminum alloy 7075, commonly used in the aerospace industry, offers superior material properties, with a Young’s modulus of E=71 GPa and Poisson ratio of *ν* = 0.33. It is often used in applications under high stress, such as aircraft spars and ground support equipment [36].

**Figure 10:**
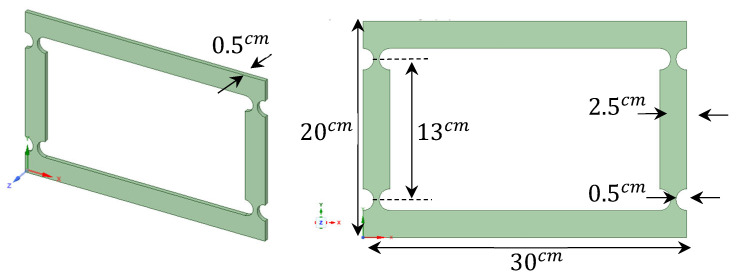
Dimensions of the four-bar geometry model (*R* = 10, *t*/*R* = 0.5).

The compliant mechanism model in Ansys was meticulously crafted to accurately represent the intricacies of the four-bar system. This model allows us to simulate and analyze the behavior of the mechanism under varying loads and conditions. A visual representation of the four-bar compliant mechanism modeled in Ansys, and a detailed view of the specific dimensions are shown in Figure 10. 

**Figure 11:**
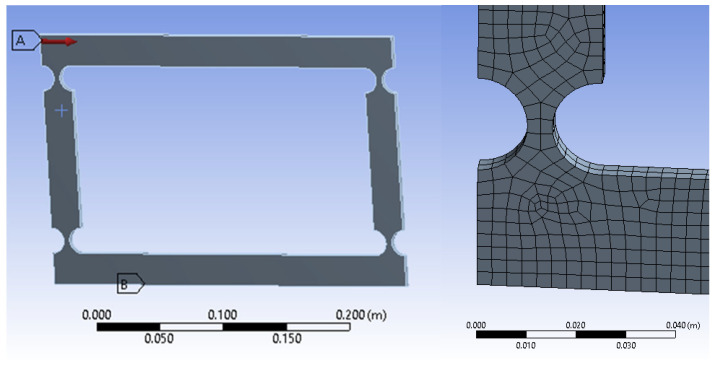
Four-bar compliant model (*r* = 10, *t*/*r* = 0.5) boundary conditions (**left**) and the FEM with quadratic quadrilateral elements (**right**).

The four-bar compliant mechanism’s geometric model and the meshed section of the CFH created in Ansys (Academic version) are shown in Figure 11 below. To define the displacement boundary conditions, the lower (bottom) segment was fully fixed, indicated by letter B, in Figure 11 (left), with zero displacements imposed in all directions. The meshing of this geometry, shown in Figure 11 (right), was performed using quadratic quadrilateral (QQ) elements. A minimum possible element size of 3 mm, allowing for changes in the thickness dimension, was used. The quadratic elements offer more accuracy than linear ones. Regarding the force boundary conditions, a single surface force, $F_{x}$, with a value ranging from 0.1 N to 200 N based on the *t*/*R* ratios, was applied to the top-left corner of the mechanism (indicated by letter A in Figure 11, left). These conditions are critical for simulating real-world scenarios and understanding how the mechanism responds to different external forces, which were used in the same method for the other FEM models of compliant mechanisms that were analyzed in this study.

#### 3.2.1. Four-Bar ABS Plastic Model

**Figure 12:**
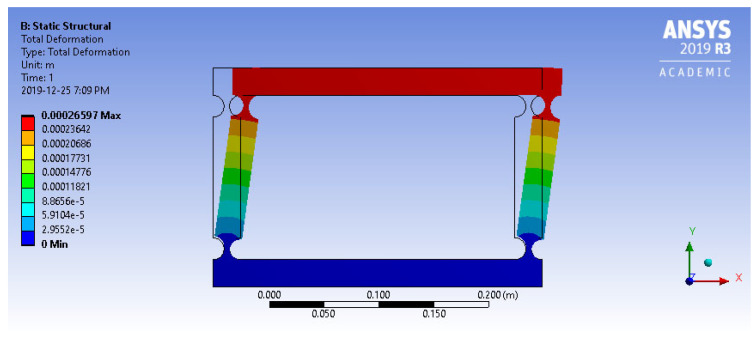
Total deformation of four-bar *ABS plastic* model (*R* = 10, *t*/*R* = 0.5) under 0.1 *N* force.

This research aimed to expand prior modeling endeavors that have effectively characterized the behavior of four-bar mechanisms constructed from both *ABS plastic* and aluminum alloy 7075. As mentioned earlier in this paper, these are the materials of choice for non-load-bearing and load-bearing structural components, respectively. In this section, the authors focus shifts to the initial modeling of the four-bar mechanism made with *ABS plastic*. Once the design specifications were established, an initial model employing *ABS plastic* was crafted and subsequently subjected to a 0.1 N load. The result, including the total deformation of the *ABS plastic* model, is illustrated in Figure 12. 

**Figure 13:**
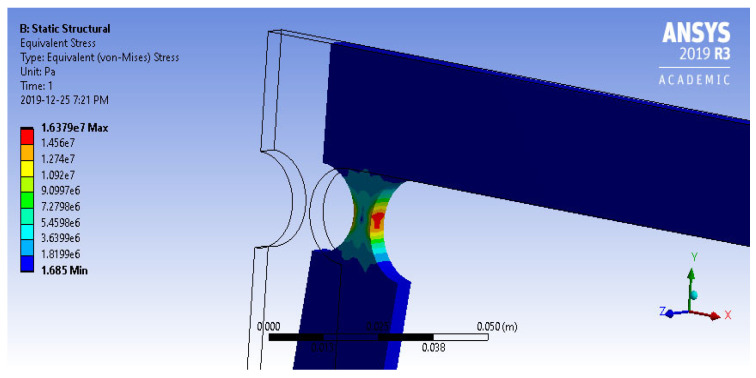
Equivalent stress of four-bar *ABS plastic* model (*R* = 10, *t*/*R* = 0.5) under a 10 N force.

The analysis of the four-bar *ABS plastic* model has yielded valuable insights into its structural behavior under loading. For instance, when subjected to a 10 N force, the mechanism experienced a maximum equivalent (Von Mises) stress of 16.37 MPa, as depicted in Figure 13 above. This stress value is well below the tensile yield strength of *ABS plastic* (29.6–48 MPa). Therefore, it can be concluded that if subjected to a 10 N force, the mechanism will exhibit a substantial margin of safety in its design (safety factors of 1.8–2.9). 

#### 3.2.2. Four-Bar Aluminum Alloy 7075 Model

**Figure 14:**
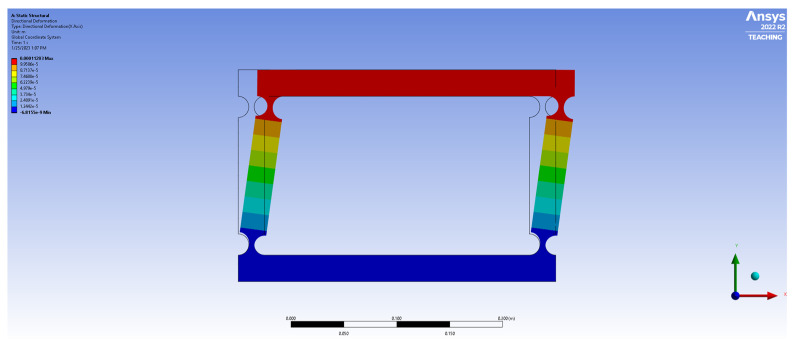
Total deformation of four-bar aluminum alloy 7075 model (*R* = 10, *t*/*R* = 0.5, *F* = 0.1 N).

**Figure 15:**
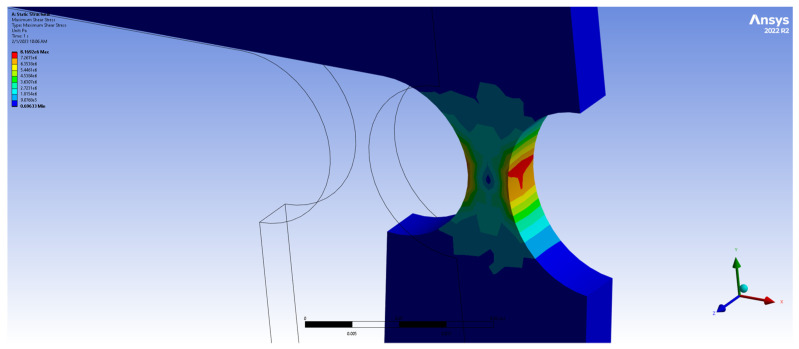
Equivalent stress of four-bar aluminum alloy 7075 model (*R* = 10, *t*/*R* = 0.5, *F* = 10 N).

In parallel with the modeling of the *ABS plastic* variant, the modeling of a four-bar mechanism constructed from aluminum alloy 7075, adhering to the same design specifications and load and displacement boundary conditions, was undertaken. The total deformation of the aluminum alloy 7075 model under a 0.1 N load was 1.12 × 10^−4^ m. Figure 14 and Figure 15, respectively, illustrate the deformation and stress distribution of the aluminum alloy 7075 four-bar model under 0.1 N and 10 N loading conditions.

**Figure 16:**
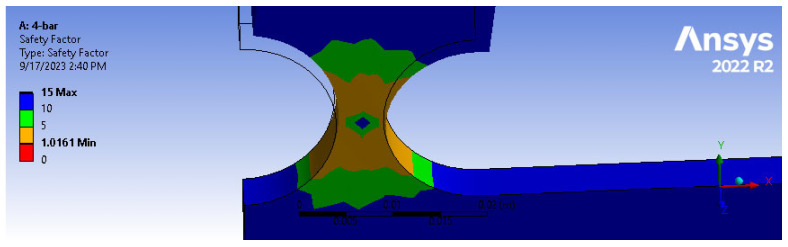
Fatigue analysis of four-bar aluminum alloy 7075 model (*R* = 10, *t*/*R* = 0.5, *F* = 10 N).

As depicted in Figure 15, when subjected to a force of 10 N, the mechanism experienced a maximum stress of 8.16 MPa, well below the material’s yield stress of 280 MPa. Furthermore, an FEM-based preliminary fatigue analysis was also performed on the same system, while subjected to a 50 N load. Figure 16 displays the safety factor (SF) calculated at various points of the mechanism, where a minimum SF of 1.0161 was observed at the hinge level (indicating a 1.6% margin of safety) and an endurance of over 1 × 10^8^ life cycles. Given that flexure hinges are often exposed to periodic loadings, fatigue analysis becomes essential. Overall, these findings proved the mechanism’s durability and reliability under the given conditions. 

#### 3.2.3. Cantilever-Bar Aluminum Alloy 7075 Model

Following the previous modeling of the four-bar mechanism, this section explores the behavior of cantilever-bar compliant mechanisms under varying loads, with a focus on the effects of different geometric parameters. Through FEA conducted in Ansys, models with different lengths and hinge dimensions were tested to gather data for accurate parameterization. Additionally, this section introduces a novel approach for developing empirical stiffness equations, paving the way for enhanced optimization of compliant mechanism design.

**Figure 17:**
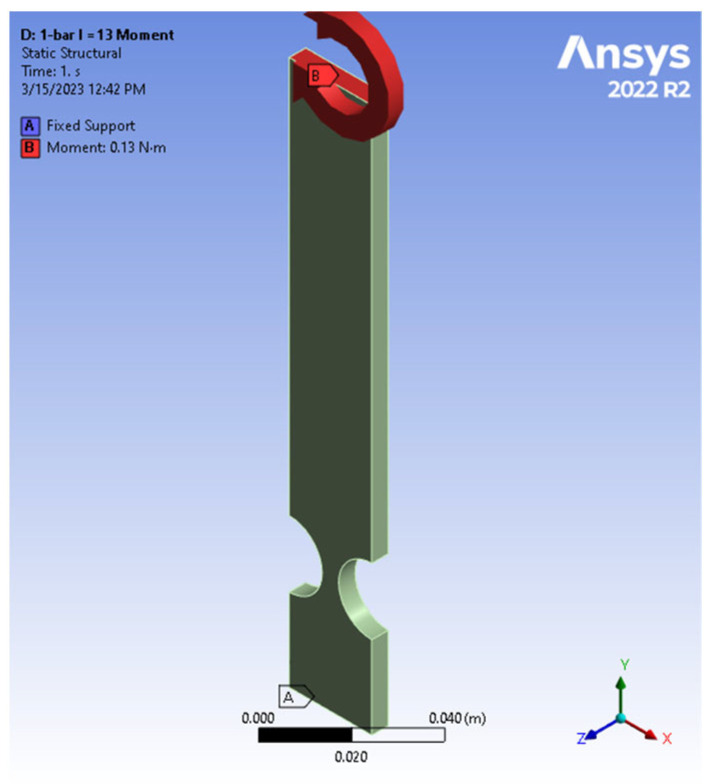
Cantilever-bar model (*R* = 10, *t*/*R* = 0.5, *L* = 13): boundary condition and moment at tip.

The cantilever bar possessed an arm length identical to the four-bar mechanism studied earlier and was subjected to a moment (refer to Figure 17). It is worth mentioning that a similar setup, subjected to an end force of an equivalent effect, was also tested. Identical end displacement and stress results were observed but the detailed comparison is omitted here for brevity. This methodology ensures consistent conditions and effectively replicates the effects of a moment on the cantilever compliant bar. Yong et al. (2008) [12] explored compliant mechanisms utilizing single bars with flexure hinges, showcasing their versatility across various forces in both the *x* and *y* directions. By introducing a compliant cantilever bar made from aluminum alloy 7075, the results of the authors’ work extend the research reported by Yong et al. (2008) [12]. The results provided valuable insights into the cantilever bar’s behavior when subjected to a tip moment, allowing for a comprehensive comparison with the four-bar compliant mechanism under a force perpendicular to the axis of the link. 

**Figure 18:**
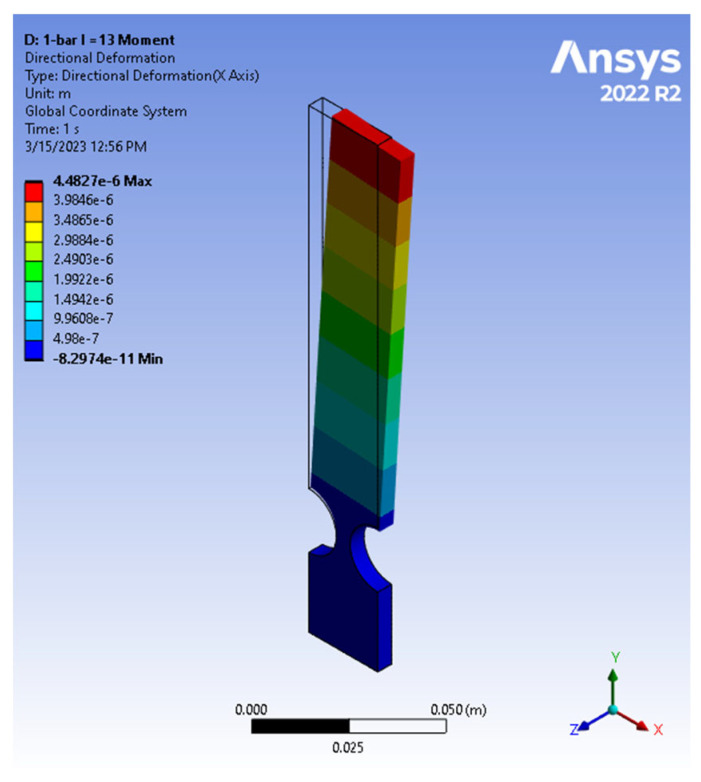
Total deformation of cantilever-bar aluminum alloy 7075 model (*R* = 10, *t*/*R* = 0.5, *L* = 13, *F* = 0.1 N).

Once the design specifications were defined, an initial aluminum alloy 7075 cantilever-bar model, with a 13 cm length, was created and subsequently subjected to tip loads of 0.1, 0.2, 1, and 2 N. The resulting deformation of the aluminum alloy 7075 cantilever-bar model is depicted in Figure 18.

**Figure 19:**
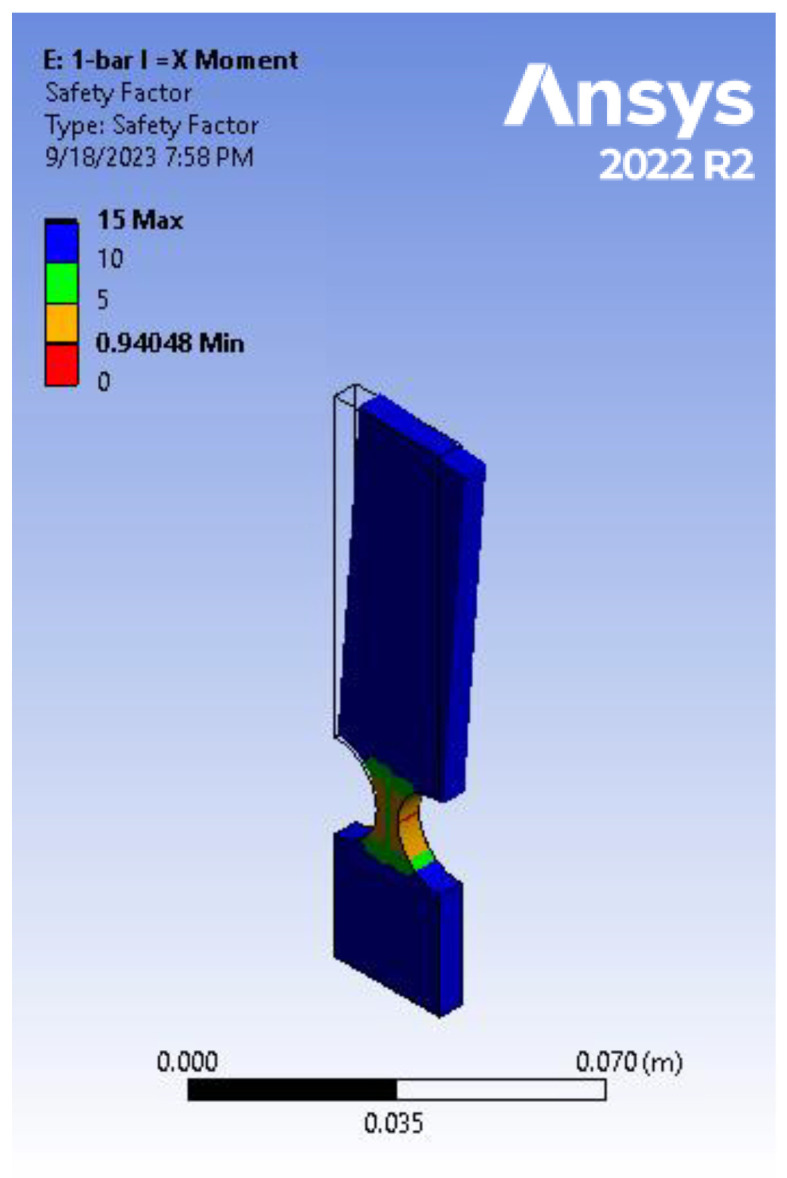
Fatigue analysis of cantilever-bar aluminum alloy 7075 model (*R* = 10, *t*/*R* = 0.5, *L* = 8 cm) (*F* = 50 N or *M* = 1.625 Nm).

Following the modeling of the previous aluminum alloy 7075 cantilever bar, a further analysis was conducted by creating another sample version of the system with a shorter length of 8 cm. This alteration aimed to create a basis for the comparison and to specifically examine the safety factor (SF), a critical aspect in mechanism design. The results of the FEA are depicted in Figure 19 alongside an image illustrating the cantilever bar subjected to 1.625 Nm moment, exhibiting the same equivalent effect as a 50 N force applied to the four-bar mechanism. This comparison sheds light on the margin of safety and performance differences between the two configurations, aiding in the optimization design of compliant mechanisms while considering fatigue concepts. 

**Figure 20:**
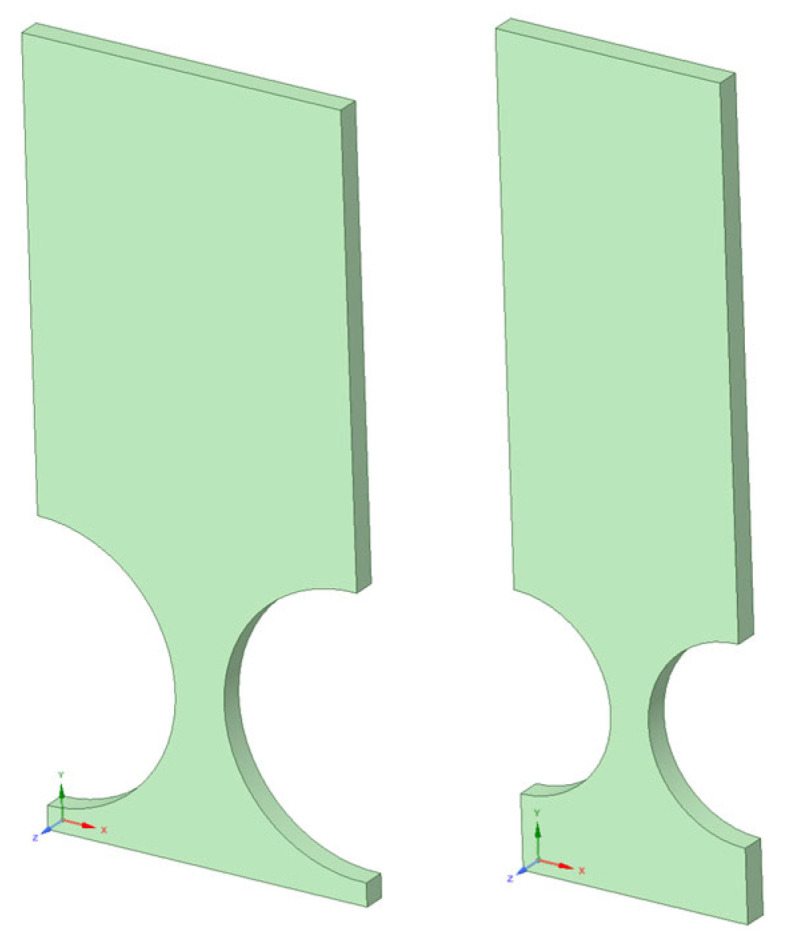
Cantilever-bar models with radiuses of 2 and 3 cm, *t*/*R* = 0.4 (*L* = 13 cm).

After analyzing various models with a fixed *t*/*R* ratio of 0.5 and adjusting both the number and length of the links, the authors’ aim was to gather a diverse range of data to ensure accurate modeling, particularly with respect to the *t* and *R* parameters. FEA-based models were developed in Ansys, incorporating three different radiuses (1 cm, 2 cm, and 3 cm), thus covering a wide range of *t*/*R* ratios spanning from 0.2 to 0.8. Figure 20 illustrates two sample models, with radiuses of 2 cm and 3 cm, and a fixed *t*/*R* ratio of 0.4. Maintaining consistent lengths from the center of the circles to the tip (end) of the cantilever bar ensured comparability with previous modeling efforts.

The FEA approach outlined in this section lays the groundwork for developing an empirical stiffness equation that encompasses a broad spectrum of circular flexure hinges (CFHs), each characterized by varying *t* and *R* geometric parameters (refer to Figure 4) falling within the range of 0.2 ≤ *t*/*R* ≤ 0.8. This methodology sets the stage for comprehensive modeling and analysis, as will be discussed in Section 5. Moreover, the strategy of modeling CFH samples with consistent lengths but differing hinge circle dimensions (*t* and *R*) holds promise for streamlining future laboratory experiments. By producing compliant cantilever bars with diverse geometric configurations, this approach offers a cost-effective solution for precise testing in laboratory settings.

### 3.3. Design Optimization Using a Hybrid PRBM–FEM Approach

A compliant mechanism design should be assessed based on specific design constraints, including the tip forces/moment, the number and length of vertical linkages, the coupler’s length, and the influences of the geometric and material (i.e., yield stress) parameters of the hinge. While the earlier analysis demonstrated the structural integrity of the mechanism under static loading, it did not account for the effects of dynamic loading that may occur in real-world applications. Drawing inspiration from the study conducted by Zhu et al. (2017) [37], where the fatigue properties of right circular flexure hinges (RCFHs) were discussed, similar aspects were explored in the current investigation. It involved subjecting test specimens to varying cyclic stress levels, which is crucial for evaluating the durability and fatigue performance of compliant mechanisms. Therefore, an Ansys-based (numerical) preliminary fatigue analysis was conducted to assess the fatigue characteristics of the compliant mechanism. To simulate the worst-case scenario, a fully reversed, constant-amplitude load was applied, alternating between maximum tension and compression of equal magnitudes. This type of loading induces the highest fatigue damage and can lead to failure even at relatively low stress levels. Using Ansys software (version 2022 R2), an *S-N* diagram (omitted here for brevity) was then generated, which provides the fatigue life (i.e., stress amplitude versus the number of cycles to failure for different load levels), endurance limit, strength, ductility exponent, as well as fatigue safety factor of the material. 

The fatigue safety factor (SF) is an important parameter used to evaluate the reliability and durability of a component or structure that undergoes cyclic loading. It shows the safety margin between the actual fatigue life of the material and the expected design life. A fatigue *SF* value below one means that the component will not last until the intended design life, whereas a higher SF represents more confidence that the component will withstand the cyclic loading without failure. For optimized design considerations, a fatigue *SF* = 1 means that the component is designed to exactly match the desired design life. The designer may, however, opt for a fatigue *SF* larger than 1 to provide a larger safety margin, depending on the specific application and desired level of reliability. The Ansys-based (numerical) fatigue study performed here investigated the *SF* for aluminum alloy 7075 subjected to fully reversed loading and a design life of 1 × 10^9^ cycles. 

**Figure 21:**
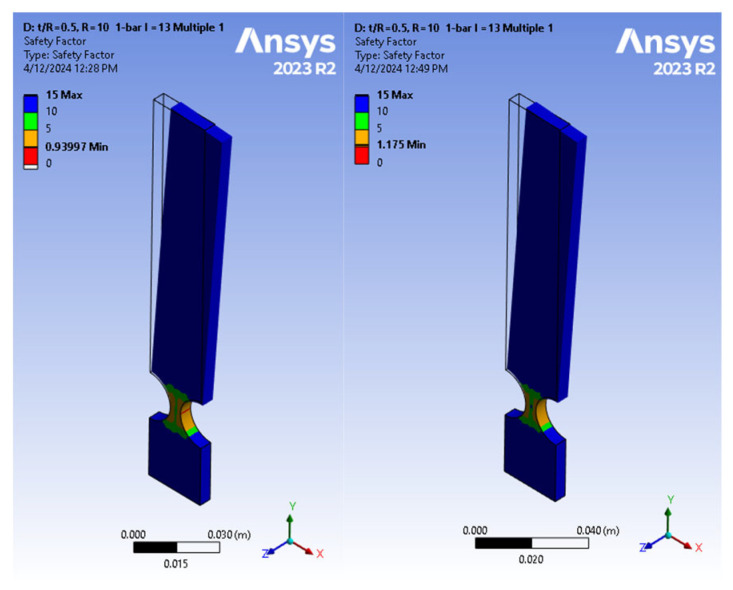
Fatigue analysis of cantilever-bar model (*R* = 10, t/R = 0.4, *L* = 13 cm): safety factor (SF); tip moment of 1.625 Nm (**left**) and 1.3 Nm (**right**).

When considering the effect of the tip force/moment on the SF, the assumptions of the isotropic nature of the material and the pseudo-rigid-body behavior predicted an inverse linear relationship for a specific hinge geometry dimension. The results (Figure 21) verified both the assumptions and Equation (2) (*R* = 10, *t*/*R* = 0.4, *L* = 13 cm). Two moments were applied, 1.625 Nm and 1.3 Nm, where a 25% decrease in the moment corresponded to a 25% increase in the SF. Figure 21 illustrates the safety factors obtained for these two moments, showing a 25% increase from 0.93997 (failure) to 1.175 (safe design). These results highlight the critical importance of considering the force/moment as a design limit to ensure an appropriate safety factor based on the application.

The comparison between the aluminum alloy 7075 four-bar and cantilever-bar models provided better insight into the effects of the number of linkages on the design parameters. Notably, when subjected to a force four times larger, the horizontal displacements in the four-bar model were observed to be equivalent to those in the cantilever bar subjected to a single force. This linear relationship in forces and deformations serves as a validation of Equations (3) and (4). For a detailed breakdown of these results, please refer to Table 3, which presents a comparison of the sample data from the two models. 

The effect of longitudinal distance between the vertical linkages (i.e., the coupler’s length, *BC*, in Figure 8) on the behavior of the four-bar compliant mechanism was also studied. It was confirmed that the distance between the vertical linkages did not affect the horizontal displacement and the mechanism’s performance remained consistent regardless of the coupler’s length, which is crucial knowledge for the design and optimization of compliant mechanisms. 

To further investigate the effects of the number of vertical linkages and the couplers’ length on the performance of the compliant mechanisms, sets of multi-bar systems were then studied. The results showed that a compliant mechanism equipped with *N* + 1 vertical linkages (i.e., a (2*N* + 1)-bar mechanism) can attain a safety factor approximately $\left(\frac{100}{N}\right)$ percent greater than a mechanism with *N* vertical linkages (i.e., a 2*N*-bar mechanism). This result, in turn, highlights the necessity of design optimization. 

**Figure 22:**
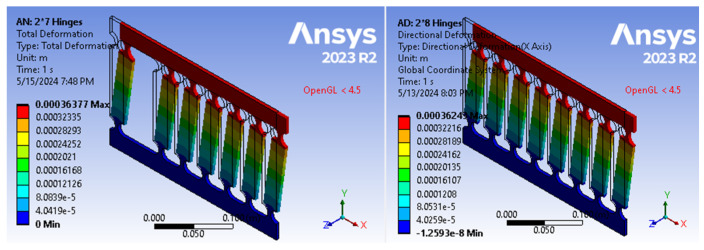
Identical horizontal displacements in multi-bar models with 7 and 8 vertical linkages (*R* = 10, *t*/*R* = 0.4, *L* = 13 cm) subjected to forces of 116.66 N and 133.33 N, respectively (SF = 1.5).

Figure 22 show the Ansys-based FEM modeling results for multi-bar compliant mechanism designs with seven and eight vertical linkages, both exhibiting identical horizontal displacement (0.00036 m, i.e., 0.36 mm) when subjected to tip forces of *F*_7_ = 116.66 N and *F*_8_ = 133.33 N (i.e., ($\frac{8\times F_{7}}{7}$)), respectively, while maintaining the same safety factor *SF* of 1.5 (with a difference of around 0.4%). This linear relationship in forces and deformation further validates Equation (5), highlighting the proportional behavior of compliant mechanisms with the number of vertical linkages. 

The impact of yield stress and forces on the maximum deflection under static testing, along with the fatigue SF, is crucial for comprehensive design evaluation. The results shown in Table 4, consistent with the findings from static force analysis, demonstrates no nonlinear behavior for the four-bar aluminum alloy 7075 mechanism. This table shows that for a hinge with dimensions of *R* = 10 and *t*/*R* = 0.5, and a moment per hinge of 1.625 Nm, a SF of 1.02 was achieved. Understanding and adhering to these design constraints are essential for optimizing compliant mechanism designs to meet specific performance requirements and reliability standards, including considerations of the maximum deflection or bending angle.

Regarding the impact of linkage length, Table 5 presents a fatigue analysis comparison among three models: the four-bar mechanism, the 13 cm cantilever-bar, and the 8 cm cantilever-bar, all subjected to a 1.625 Nm moment per hinge (equivalent to a 50 N force applied to the four-bar mechanism). Despite nearly identical bending angles observed in these models, the four-bar mechanism achieved a safety factor (SF) of 1.02, while both the 13 cm and 8 cm cantilever-bars experienced failure (with an SF of 0.94).

**Figure 23:**
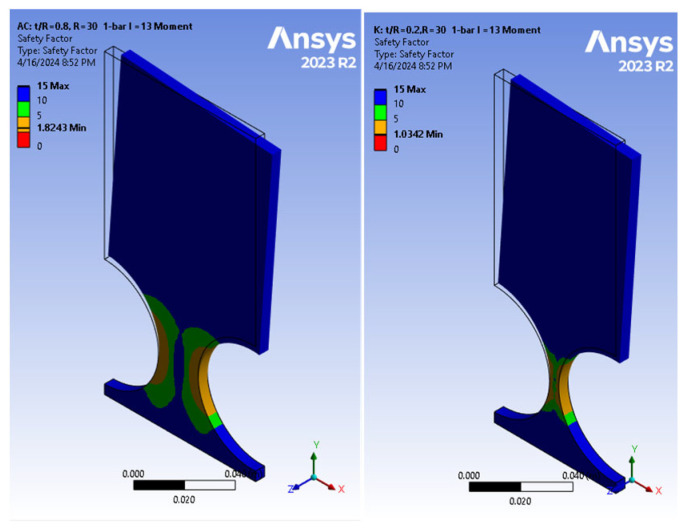
Fatigue analysis of cantilever-bar model. (**left**) *M* = 16.25 Nm, *t*/*R* = 0.8, *SF* = 1.82; (**right**) *M* = 1.625 Nm, *t*/*R* = 0.2; *L* = 13 cm, *SF* = 1.03.

Continuing the investigation into the parameters contributing to the optimization design process, an FEA on the cantilever bar in Ansys was conducted to explore parameters such as *t* and *R*. These FEA-based models included three different radiuses (1 cm, 2 cm, and 3 cm), encompassing a wide range of *t*/*R* ratios ranging from 0.2 to 0.8. Figure 23 demonstrates how increasing the *t*/*R* ratio at a fixed radius of 30 cm led to a reduction in the SF at the hinges, from 1.82 to 1.03, providing a promising path for optimizing the design to ensure safety during fatigue tests and reducing weight, resulting in cost savings.

#### 3.3.1. Comparison of Compliance/Stiffness Results with FEA Results

**Figure 24:**
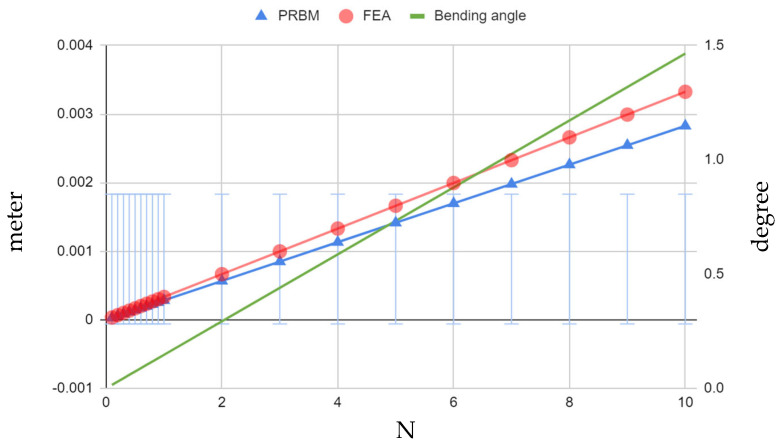
Four-bar *ABS plastic* model (*R* = 10, *t*/*R* = 0.5)—Paros PRBM [14] vs. FEA displacement and bending angle.

Through a thorough analysis, encompassing both *ABS plastic* and aluminum alloy 7075 models, the behavior of both four-bar and cantilever-bar mechanisms subjected to a range of tip forces (0.1 N to 10 N) was investigated. The current equations in the PRBM method, discussed earlier in previous sections and summarized in Table 1, were used to calculate the displacement and bending angles caused by the range of forces applied to these mechanisms. These calculated displacements are very important as they demonstrate the linearity of the mechanisms’ behavior. It is important to point out that (for the *ABS plastic* model), there was a high −17.60% error between the displacements calculated from the PRBM equation presented by Paros [14] and those obtained from Ansys-based FEM simulations (Figure 24). The bending angle for each static tip force applied was also determined, as outlined in Figure 24. When subjected to forces ranging from 0.1 *N* to 10 *N*, the system exhibited a noteworthy range of bending angles, spanning from 0.01465 to 1.46495 degrees for a hinge with dimensions of *R* = 10 and *t*/*R* = 0.5, (refer to Figure 10 for other parameters). This extensive range of deformation underscores the inherent flexibility and compliance of *ABS plastic*, enabling significant bending in response to diverse loads. 

While engineering applications often require greater bending angles, the present study provides insights into the linear behavior of compliant mechanisms within the limitations of linear flexible beam equations. To address the need for larger deformations, one could potentially scale up the results by integrating FEA, PRBM, and nonlinear beam theories, and obtain more accurate estimations of large deformations (with reduced errors) through the development of relevant empirical equations. In addition, linear displacements can be scaled up by integrating, for example, a pantograph-type mechanism into the 2*N*-bar systems discussed in this paper. 

**Figure 25:**
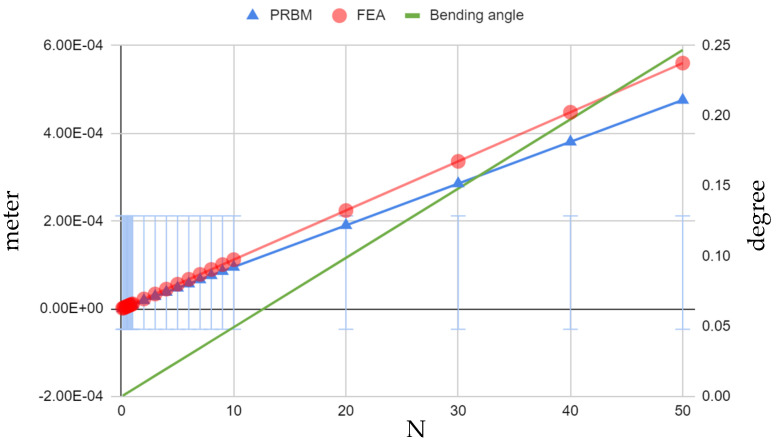
Four-bar aluminum alloy 7075 model (*R* = 10, *t*/*R* = 0.5)—Paros PRBM [14] vs. FEA displacement and bending angle.

Referring to the findings in the case of the aluminum alloy 7075 model, when comparing the PRBM results to those of the FEM simulations conducted using Ansys, similar errors of −17.71% were observed (Figure 25). This level of deviation from the FEM simulation data is clearly not within acceptable limits and necessitates a thorough reevaluation of our analysis methodologies. 

Nonetheless, following obtaining accurate bending angles using the PRBM method, the graphs presented in Figure 25 provide a comprehensive dataset encompassing the bending angles under varying force levels. In contrast to *ABS plastic*, as expected, the aluminum alloy 7075 four-bar mechanism exhibited a significantly narrower range of bending angles, spanning from 0.000493 to 0.049375 degrees under the same force range due to the much higher elastic modulus of the aluminum alloy 7075. 

The examination of the aluminum alloy 7075 model also provided crucial insights into the structural integrity and safety of the mechanism. The consistent observation that the maximum stress within the mechanism remained well below the material’s elastic limit of 280 MPa is proof of its durability and reliability. The FEM-based Ansys analysis led to a safety factor of 1.02 under a 50 N force application, with a maximum stress of only 81.4 MPa, as depicted in Figure 25, while exhibiting a maximum bending angle of 0.25 degrees.

In conclusion, the investigation into material properties, including a comparative analysis of aluminum alloy 7075 and *ABS plastic* modeling results, alongside fatigue analyses to evaluate design constraints, has provided valuable insights. The observed linear relationship between elastic modulus (*E*) and the compliance results paves the way for using other linearly elastic materials, which offer significantly higher elasticity and potentially greater deflections or bending angles—essential qualities for compliant mechanism design. Additionally, the assessment of the number of linkages, the effect of yield stress and forces, the impact of linkage length, and geometric hinge parameters underscored the significance of integrating additional linkages perpendicular to the applied force direction or converting a cantilever-bar into a four-bar mechanism, and subsequently to a multi-bar mechanism. As also stated earlier, this integration, through, for example, adding one vertical link to a 2*N*-bar mechanism, can potentially enhance the safety factor, increasing it by $\left(\frac{100}{N}\right)$ percent. However, it is essential to acknowledge that increasing the number of bars leads to a proportional increase in the system’s weight. These findings highlight the complex trade-offs inherent in compliant mechanism design and emphasize the need for thoughtful optimization to achieve the desired performance outcomes.

## 4. Empirical Rotational Stiffness/Compliance Equation ($∆\alpha_{z}/M_{z}$)

In the analysis of compliant mechanisms, it is crucial to address the limitations of certain analytical models in accurately predicting the behavior of structures. In the context of the cantilever-bar design, while the present study’s results echo the linearity of stiffness equations presented by Paros and Weisbord (1965) [11], a substantial 17.7% difference between the predicted and FEM simulation results using the Pseudo-Rigid-Body Model (PRBM) were found. This underscores the necessity of employing more sophisticated methods to capture the complexities of compliant structures. Notably, a study by Yong et al. (2008) [12] similarly emphasized the inadequacy of the existing analytical models, particularly in the tip displacements in the *x*-direction, where significant errors were observed. To overcome such challenges, finite element analysis (FEA) emerges as a powerful tool, providing a more accurate depiction of displacement and stiffness in compliant mechanisms. The graphical representation of these errors, as shown in Figure 24 and Figure 25, emphasizes the advantage of FEA over traditional analytical models, urging the adoption of such advanced techniques for precise and reliable design processes.

In the literature review presented earlier in this paper, the accuracy of various compliance models for CFHs were compared with the benchmark of finite element results and previously existing empirical compliance models that can be used for a wide range of geometric parameters. The empirical equations derived from FEA are proven and preferred solutions because many of the existing analytical formulas cannot accurately estimate the compliance characteristics of flexure hinges for a wide range of geometric parameters. The examination of parameters such as *t* and *R* and how they affect the stiffness/compliance of the mechanism revealed the need for careful consideration to develop an empirical equation for torsional stiffness/compliance.

As highlighted in the preceding section, to the best of authors’ knowledge, no accurate design equations (within a 5% error) for rotational stiffness/compliance (denoted by $∆\alpha_{z}/M_{z}$) currently exist in the open literature [12]. Consequently, in what follows, using the regression method and the results obtained from the FEA, a new general empirical equation for rotational stiffness/compliance, $K_{\alpha_{z}}$, was formulated for 0.2 ≤ *t*/*R* ≤ 0.8. To this end, FEM models were created in Ansys, with three different radiuses of 1, 2, and 3 cm, covering various *t*/*R* ratios ranging from 0.2 to 0.8. A tip moment ($M_{z}$) was applied to each model, and the corresponding bending angle deformations ($∆\alpha_{z}$) were recorded. Multiple polynomial regression functions with higher-order terms were used to fit the data points, leading to the following empirical stiffness Equation (6).

(6) $$k_{\alpha_{z}}=Ew\;\left[\begin{array}{c} C_{0}+C_{1}\;\left(R^{-1}\;\right)+C_{2}\;\left(t^{2}\right)\;\\ +C_{3}\;\left(R\cdot t\right)+C_{4}\;\left({\left(R\cdot t\right)}^{2}\right)\;\\ +C_{5}\;\left(R\cdot t^{2}\right)+C_{6}\left(t^{3}\right)\;\;\;\\ +C_{7}\left({\left(t+R\right)}^{2}\right)\cdot \;t^{2})\; \end{array}\right]$$

The coefficients $C_{0}\;through\;C_{7}$ are shown in Table 6 below. The other parameters that appear in the equation are shown in Figure 4.

**Figure 26:**
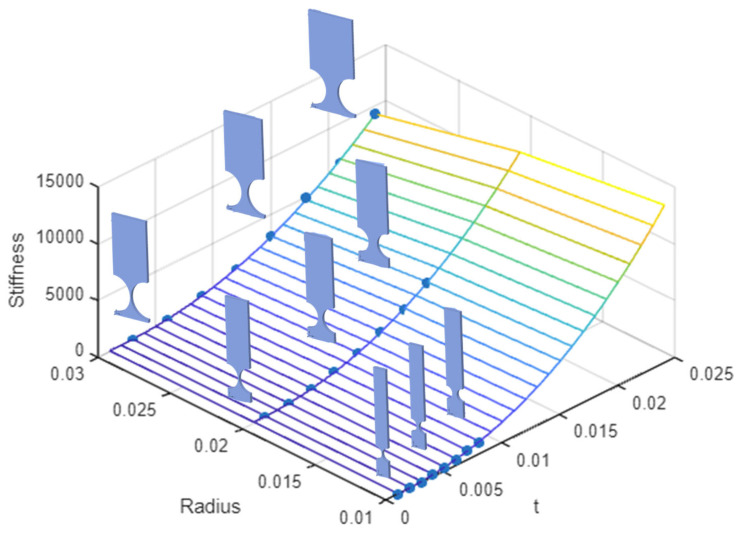
Comparison of empirical regression model results for rotational stiffness/compliance ($∆\alpha_{z}/M_{z}$) of CFHs with FEA results, showing variability in hinge geometry.

**Figure 27:**
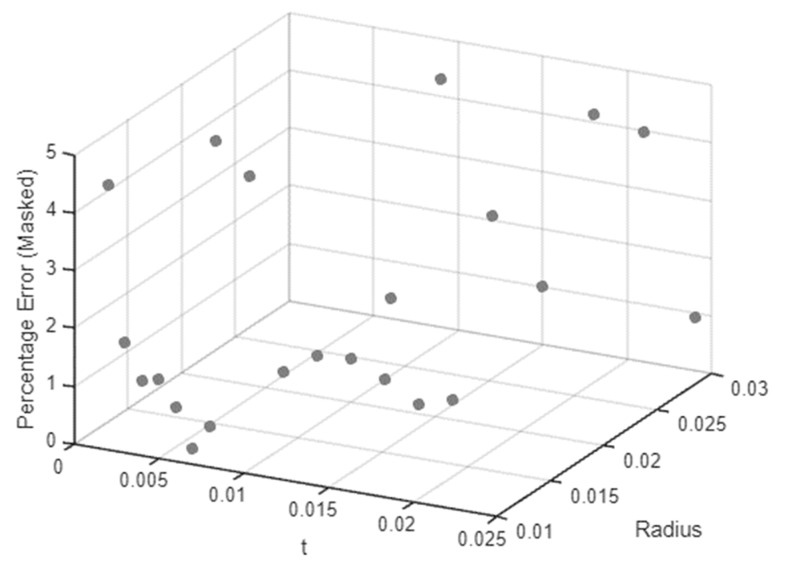
Threshold percentage error between the empirical and actual stiffness values.

The plotted results from Equation (6) shown in Figure 26 depict the regression model for the rotational stiffness/compliance of CFH hinges compared with the results obtained from the FEA; they illustrate the variability in geometry across different hinges. Figure 27 shows the graph of the percentage error (thresholded), indicating the difference between the regression model and the actual FEA data. Higher-order terms were included for $K_{\alpha_{z}}$ to reduce errors. The error range goes from 0.149% as the lowest value to 4.58% as the highest value, with an average error of only 1.74%, showing a reliable and fairly accurate fit. Considering the team’s future research developments, it would be advisable to also verify the presented empirical model experimentally, through laboratory tests on various linearly elastic materials. This would provide further insights into the materials’ performance and enhance the study’s overall value. 

## 5. Conclusions

A detailed analysis of compliant mechanisms, focusing on *ABS plastic* and aluminum alloy 7075 in both four-bar and cantilever-bar configurations, was presented. The investigation highlighted the flexibility and compliance of the *ABS plastic* four-bar mechanism, showcasing its capacity to withstand various loads. Despite discrepancies between the PRBM calculations and FEM-based Ansys simulations, its predictable linear behavior and fatigue testing results affirm its suitability for diverse applications.

Comparing cantilever-bar, four-bar, and multi-bar models, a linear relationship between deformation and applied force was observed, emphasizing the importance of the number of vertical linkages for higher safety factors. When compared to a 2*N*-bar (with *N* vertical bars), a 2(*N* + 1)-bar compliant mechanism demonstrated a ($\frac{100}{N}$) percent higher safety factor, but it comes with increased system weight, requiring careful consideration. Determining the maximum achievable bending angle guides designers, promoting insights into optimal configurations. While replacing conventional torsional springs in the Pseudo-Rigid-Body Model (PRBM) with equivalent compliant ones offers innovation and known advantages, vigilance about design constraints is crucial. Evaluating the design limits empowers designers to make informed decisions, considering the material’s properties, hinge count, and dimensions. The study addresses the need for innovative solutions in precise modeling, particularly for flexure hinges with variable cross-sections. 

In conclusion, the present study marks an advancement in the accuracy of compliant mechanism design through the integration of PRBM and finite element methods. The focus on developing a new empirical polynomial torsional (or rotational) stiffness/compliance equation aimed to address the geometric parameter variations and employed material, providing valuable insights for tailored designs. The validation against FEA demonstrated a generally accurate prediction of the results, with an average error of 1.74% for 0.2 ≤ *t*/*R* ≤ 0.8, establishing the effectiveness of the authors’ approach. This contribution opens new avenues for enhanced precision in compliant mechanism design, ensuring reliability and efficiency in various practical applications. 

## Figures and Tables

**Table 1:** Compliance/Stiffness Equations of Circular Flexure Hinges for Specific *t*/*R* Ranges, proposed by Yong et al. [12] with updates based on the present study (see Section 4).

Research Team	∆*α_z_*/*M_z_*	% Difference			∆*y*/*F_y_* (with SC)	% Difference			∆*x*/*F_x_*	% Difference		
	*t*/*R* Range	Min.	Max.	ave.	*t*/*R* Range	Min.	Max	ave.	*t*/*R* Range	Min.	Max	ave.
Paros (full) [14]	0.05 ≤ *t*/*R* ≤ 0.1	1.8	5.0	3.5	0.05 ≤ *t*/*R* ≤ 0.1	2	4	3.1	0.25 ≤ *t*/*R* ≤ 0.65	0.3	4.9	2.4
Paros (simple) [8]	0.05 ≤ *t*/*R* ≤ 0.2	1.2	4.9	3.1	0.05 ≤ *t*/*R* ≤ 0.1	3	5.6	4.3	Not recommended			
Lobontiu [15]	0.05 ≤ *t*/*R* ≤ 0.1	1.8	5.0	3.5	0.05 ≤ *t*/*R* ≤ 0.1	2	3.9	2.9	0.25 ≤ *t*/*R* ≤ 0.65	0.3	4.9	2.4
Wu & Zhou [16]	0.05 ≤ *t*/*R* ≤ 0.1	1.8	5.0	3.5	0.05 ≤ *t*/*R* ≤ 0.1	2	4	3.1	0.25 ≤ *t*/*R* ≤ 0.65	0.3	4.9	2.4
Tseytlin [17]	0.4 ≤ *t*/*R* ≤ 0.6	0.7	4.5	2.5	NA	NA	NA	NA	NA	NA	NA	NA
Smith [18]	0.2 ≤ *t*/*R* ≤ 0.65	0.8	3.7	2.4	NA	NA	NA	NA	NA	NA	NA	NA
Schotborgh [19]	0.05 ≤ *t*/*R* ≤ 0.65	0.03	2.5	1.2	NA	NA	NA	NA	NA	NA	NA	NA
Yong [12]	NA	NA	NA	NA	0.05 ≤ *t*/*R* ≤ 0.8	0	2.7	0.07	0.05 ≤ *t*/*R* ≤ 0.8	0	1.1	0.08
Present study	0.2 ≤ *t*/*R* ≤ 0.8	0.15	4.58	1.7	NA	NA	NA	NA	NA	NA	NA	NA

**Table 2:** Circular flexure hinge dimensions, *t*/*R* ratio = 0.5.

Parameter	Dimension (mm)
Width (*w*)	5
Hinge thickness (*t*)	5
Hinge radius (*R*)	10

**Table 3:** Four-bar vs. cantilever-bar aluminum alloy 7075 model (*R* = 10, *t*/*R* = 0.5): FEA displacement.

Four-Bar Model	Cantilever-Bar Model	Displacement
Force (N)	Force (N)	∆x (m)
0.4	0.1	4.48.33 × 10^−6^
0.8	0.2	8.96.48 × 10^−6^
4	1	4.48 × 10^−5^
8	2	8.96.48 × 10^−5^

**Table 4:** Four-bar aluminum alloy 7075 model (*R* = 10, *t*/*R* = 0.5): displacement, stress, safety factor, and bending angle.

$\frac{Moment(N.m)}{hinge}$	Safety Factor (SF)	Displcement (m)	Bending Angle (Degrees ^o^)	Max Stress (von Misses)
0.325	5.08	1.12 × 10^−4^	0.049375	8.17 × 10^6^
0.650	2.54	2.24 × 10^−4^	0.098755	3.26 × 10^7^
0.975	1.69	3.36 × 10^−4^	0.148135	4.89 × 10^7^
1.300	1.27	4.48 × 10^−4^	0.197515	6.51 × 10^7^
1.625	1.02	5.60 × 10^−4^	0.246890	8.14 × 10^7^

**Table 5:** Comparison of fatigue analysis results from four-bar and two cantilever-bar models (*R* = 10, *t*/*R* = 0.5) with different lengths (1.625 Nm/hinge).

Four-Bar Model	Cantilever-Bar Model	Cantilever-Bar Model
${L=13}^{\left(cm\right)}$	${L=13}^{\left(cm\right)}$	${L=8}^{\left(cm\right)}$
1.0161 (safe)	0.9403 (failure)	0.9405 (failure)

**Table 6:** Coefficients for equation for empirical polynomial stiffness ($K_{\alpha_{z}}$).

$C_{0}$	$C_{1}$	$C_{2}$	$C_{3}$	$C_{4}$	$C_{5}$	$C_{6}$	$C_{7}$
2.1476 × 10^−7^	−1.6661 × 10^−9^	0.0386	−0.0056	−19.4348	0.9374	1.5832	−12.0564

## Data Availability

All data generated or analyzed during this study are included within the article.
